# The impact of short hospital stay on prognosis after acute myocardial infarction: An analysis from the ACSIS database

**DOI:** 10.1002/clc.23652

**Published:** 2021-05-26

**Authors:** Orr Tomer, David Leibowitz, Michal Einhorn‐Cohen, Nir Shlomo, Idit Dobrecky‐Mery, Alex Blatt, Simcha Meisel, Ronny Alcalai

**Affiliations:** ^1^ The Heart Institute, Hadassah Medical Organization and Faculty of Medicine Hebrew University of Jerusalem Jerusalem Israel; ^2^ Neufeld Cardiac Research Institute Sheba Medical Center Ramat Gan Israel; ^3^ Department of Cardiology Bnai Zion Medical Center Haifa Israel; ^4^ Department of Cardiology Kaplan Medical Center and Hebrew University Rehovot Israel; ^5^ Heart Institute Hillel Yaffe Medical Center Hadera Israel

**Keywords:** acute coronary syndrome, length of hospital stay, myocardial infarction

## Abstract

**Background:**

Current evidence regarding the optimal length of hospital stay (LOS) following myocardial infarction (MI) is limited. This study aimed to examine LOS policy for MI patients and to assess the safety of early discharge.

**Methods:**

A prospective observational study that included patients with STEMI and NSTEMI enrolled in the Acute Coronary Syndrome Israeli Survey (ACSIS) during the years 2000–2016. Patients were divided into three subgroups according to their LOS: <3 days (short‐LOS), 3–6 days (intermediate‐LOS) and >6 days (long‐LOS). We compared baseline characteristics, management strategies and clinical outcomes at 30 days and 1 year in these groups.

**Results:**

Ten thousand four hundred and fifty eight patients were enrolled in the study. The LOS of MI patients gradually decreased over time. Short‐LOS and intermediate‐LOS patients had similar clinical characteristics while patients in the long‐LOS group were older with more co‐morbidity. There was no difference in the clinical outcomes, including re‐MI, arrhythmias, 30 days MACE, and 30 days mortality between the short‐LOS and intermediate‐LOS groups. However, the rate of re‐hospitalizations was higher in the short‐LOS group (20.9% vs. 17.8%, *p* = .004) without evidence of increased cardiovascular events. In multivariate analysis, the LOS did not predict either 30 days mortality (HR: 1.3; CI:0.45–5.48), nor MACE at 30 days (HR: 1.1; CI:0.79–1.56).

**Conclusion:**

Our study suggests that an early discharge strategy of up to 3 days from admission is safe for low and intermediate‐risk patients after both STEMI and NSTEMI. Nevertheless, this strategy is associated with an increased risk of potential avoidable readmission and there might be psychological and social factors that may warrant a longer stay.

## 1 INTRODUCTION

Until the 1950s, patients with myocardial infarction (MI) were treated by immobilization and prolonged hospital stay (4–6 weeks).^1^, ^2^, ^3^ This policy gradually changed in the following decades with a median hospitalization of 21 days in 1970, 14 days in 1980, and less thereafter.^4^ Improvement in the management of acute MI over the past several decades, in conjunction with a policy of early mobilization, has led to a dramatic decline in overall mortality with a steady decrease in the length of hospital stay (LOS).^5^, ^6^, ^7^ Though decreased LOS provides a significant cost savings for health care systems, there is concern that this policy might put patients at risk due to premature discharge.

The available evidence regarding the optimal LOS after a MI is scarce. The guidelines of the European Society of Cardiology^8^ currently recommend that the optimal LOS should be determined on an individual basis, according to the patient's cardiac risk, comorbidities, functional status, and social support.

Several studies have shown that patients with STEMI who underwent successful primary PCI and complete revascularization can be safely discharged from hospital within 48–72 h.^3^, ^9^, ^10^, ^11^, ^12^, ^13^, ^14^ These low‐risk patients can be recognized using various risk score systems^9^, ^10^, ^11^, ^12^, ^13^ such as the Second Primary Angioplasty in Myocardial Infarction (PAMI‐II) criteria^9^ or the Zwolle primary PCI Index.^10^, ^11^, ^12^, ^13^

Only a few studies which investigated the safety of early discharge^9^, ^11^, ^12^, ^15^, ^16^ post MI were randomized and most of them were relatively small and underpowered. Moreover, there are no actual guidelines recommendations concerning the optimal LOS following NSTEMI.^17^, ^18^, ^19^

In the present study, we used the large ACSIS (Acute Coronary Syndrome Israeli Survey) database, in order to assess whether an early discharge strategy is safe, and to define the characteristics of patients who could benefit the most from this strategy.

## 2 METHODS

### 2.1 Setting

An observational study using the Acute Coronary Syndrome Israeli Survey (ACSIS) database. The ACSIS is a biannual nationwide survey performed since the year 2000 that includes all ACS patients admitted during a 2‐month period to the intensive cardiac care units and cardiology wards of 26 public Israeli hospitals. This survey was approved by the Institutional Review Boards of each site and all patients signed a written informed consent. The survey prospectively collects pre‐specified data on patients admitted with the diagnosis of ACS including unstable angina, non‐ST‐segment elevation myocardial infarction (NSTEMI) and ST‐segment elevation myocardial infarction (STEMI). Experienced nurses and study coordinators collect follow‐up information at 30 days. Mortality at 1 year is obtained from the Israeli National Population registry. The present analysis included all patients with acute MI (STEMI or NSTEMI) who participated in the survey throughout 2000–2016. Patients who died during hospitalization and those who underwent CABG were excluded. The ACSIS survey was approved by the Institutional Review Boards of each site.

### 2.2 Design

All patients were segregated according to their LOS (LOS): up to 3, 3–6, and over 6 days. The short LOS was chosen based on previous studies and guidelines of early discharge after STEMI.^8^, ^9^, ^11^, ^12^ The intermediate LOS was chosen arbitrary representing the next 72 h (3–6 days). The long LOS was all other patients. We compared the groups for baseline demographic and clinical characteristics, management strategies during the index event, and outcome at 30 days and mortality at 1 year. Outcomes included all‐cause mortality, MI, recurrent angina or revascularization (defined as new unplanned need for percutaneous coronary intervention or coronary artery bypass grafting surgery), arrhythmias and re‐hospitalization from any cause. Major adverse cardiovascular event (MACE) was defined as the composite of death, unstable angina, MI/ischemia, cerebrovascular event, stent thrombosis and an unplanned need for revascularization procedure.

Additional comparisons were made between STEMI and NSTEMI patients and according to the GRACE‐score (Global Registry of Acute Coronary Events Risk Score) tertiles.

### 2.3 Statistical analysis

Differences between groups were tested with chi‐square for categorical variables and with *t*‐test or Mann–Whitney test as appropriate for normally or non‐Gaussian distributed continuous variables. Logistic regression, presented as a forest plot, was calculated to assess the relationship between baseline characteristics and LOS to the risk of re‐hospitalization.

Survival curve plots were conducted using Kaplan–Meier estimator and differences between survival curves were tested using the Log‐rank test. In order to explore the effect of LOS on survival or on 30 days MACE, logistic or Cox models adjusted for selected covariates were utilized.

Statistical analyses were performed using R Core Team (2015).^20^ Statistical significance was defined as *p*‐value <.05.

## 3 RESULTS

### 3.1 Trends

A total of 13 438 patients were included in the study, of which, 1703 patients were in the short LOS (<3 days), 7880 in the intermediate LOS (3–6 days), and 3855 in the long LOS (more than 6 days) categories. Hospital stay for MI has significantly shortened throughout the years (Supplementary Figure 1 shows the percentage of patients in each LOS group). In 2000, most patients were hospitalized for more than 6 days (>50%), and only a small percentage (<5%) was discharged early, whereas by 2016 more than a quarter of patients were discharged very early and the number of long hospitalizations dropped significantly (<20%).

### 3.2 Baseline and clinical characteristics

**FIGURE 1:**
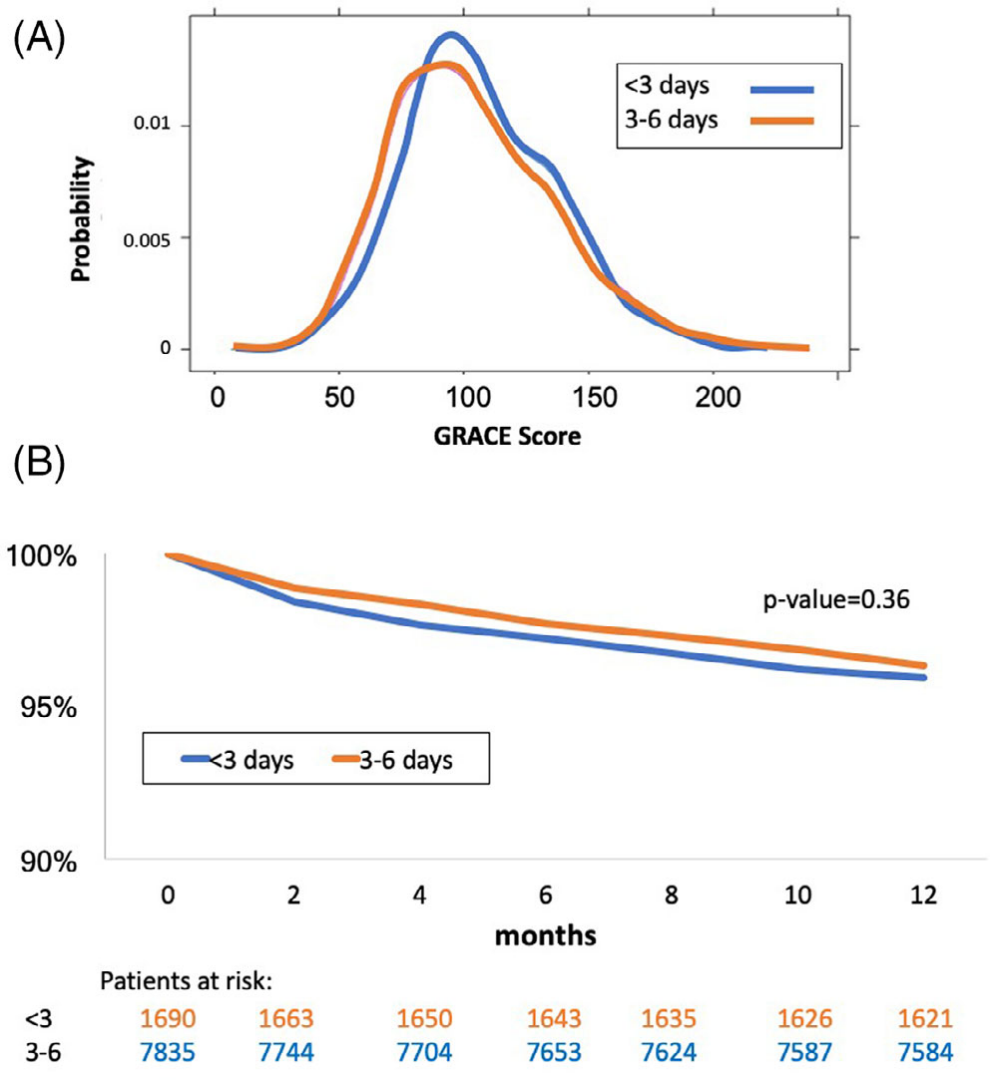
(A**)** Global Registry of Acute Coronary Events Risk (GRACE) risk score probability densities of patients in the intermediate‐length of hospital stay (LOS) and short‐LOS groups (data available for *N* = 6160). Distribution was not significantly different (*p* = .092). (B**)** One–year Kaplan‐Mayer survival curves of the short and intermediate length of hospital stay groups

Baseline characteristics of the three groups are shown in Table 1. Patients in the long LOS group (>6 days) were older with more co‐morbidities and risk‐factors than patients in the intermediate and short LOS groups. The two latter groups were comparable, with a similar GRACE risk score (Table 1 and Figure 1A). Further analysis focused on the short and intermediate LOS groups (LOS <3 and 3–6 days) since the hospital duration of the long LOS group was driven by clinical indications and not by routine policies.

**TABLE 1:** Baseline characteristics of the three LOS groups

Hospitalization duration (days)			
	Short (<3)	Intermediate (3–6)	Long (>6)
Number of patients	1703	7890	3865
Baseline characteristics			
Age, years (mean ± SD)	61.39 ± 12.31	61.94 ± 12.69	66.77 ± 12.94^a^
Gender (male)	1397 (82.0)	6265 (79.4)^b^	2768 (71.6)^a^
Higher education/academic	189 (29.3)	753 (32.0)	138 (23.4)^a^
Marital status: married/attached	865 (83.5)	3181 (79.5)^b^	958 (72.9)^a^
Dyslipidemia	1227 (72.3)	5187 (65.9)^b^	2298 (59.6)^a^
Hypertension	1042 (61.4)	4384 (55.7)^b^	2339 (60.7)^a^
Current smokers	663 (39.1)	3231 (41.2)	1174 (30.6)^a^
Diabetes mellitus	609 (35.8)	2534 (32.2)^b^	1507 (39.1)^a^
Family history of CAD	472 (29.9)	2094 (28.4)	713 (19.3)^a^
BMI (kg/m^2^), (mean ± SD)	28.29 ± 11.23	27.92 ± 8.99	27.95 ± 12.86
Prior IHD	829 (48.7)	2946 (37.3)^b^	1596 (41.3)^a^
History of CHF	88 (5.2)	452 (5.7)	432 (11.2)^a^
Chronic renal failure	131 (7.7)	638 (8.1)	591 (15.3)^a^
PVD	96 (5.7)	525 (6.7)	441 (11.4)^a^
s/p CVA/TIA	101 (5.9)	504 (6.4)	398 (10.3)^a^
ECG pattern: ST elevation	451 (26.5)	3726 (47.3)^b^	1937 (50.3)^a^
Grace score>140	110 (12.3)	656 (12.9)	552 (27.2)^a^

Abbreviation: CAD, Coronary Artery Disease, CHF, Congestive Heart Failure, LOS, length of hospital stay, CVA, Cerbro Vascular Accident, IHD, Ischemic Heart Disease, PVD, Peripheral Vascular Disease, s/p, status post, TIA, Transient Ischemic Accident..

^a^ *p*<.05, comparison between all three groups. Brackets represent percentage.

^b^ *p*<.05, intermediate‐LOS compared to the short‐LOS (<3 days) group.

The short LOS group had a significantly higher percentage of male and married subjects compared to the intermediate LOS group. Interestingly, there was a higher proportion of patients with risk factors, such as dyslipidemia, hypertension, and diabetes mellitus and known ischemic heart disease in the short LOS group. Other co‐morbidities, such as smoking, renal failure or heart failure, were similar between groups.

Comparison of clinical characteristics (Supplementary Table 1) reveals that NSTEMI, normal ejection faction, non‐significant coronary disease and radial access were more frequent in the short‐LOS group. In hospital complication rate was low in both groups but still more frequent in the intermediate LOS group compared with the short LOS group (Supplementary Table 1).

### 3.3 Outcomes

Major clinical outcomes in the short and intermediate LOS groups are shown in Table 2. The rate of re‐hospitalization was higher in the short‐LOS group. All other outcome endpoints including MACE and mortality were not different between groups. These results were similar when sub‐divided into STEMI and NSTEMI patients (data not shown), or when sub‐divided between early period (2000–2008) and late period (2010–2016) (Supplementary Table 2). Interestingly, re‐hospitalization was significantly higher only in the early period.

**TABLE 2:** Clinical outcome and mortality rates at 30 days

Hospitalization duration (days)			
	<3	3–6	*p* value
Number of patients	1703	7890	
Re‐hospitalization	330 (20.9)	1330 (17.8)	**.004**
RE‐MI	19 (1.2)	95 (1.3)	.716
Angina	42 (3.8)	191 (4.0)	.822
Arrhythmia	7 (0.39)	33 (0.3)	.423
30‐day MACE	120 (7.0)	640 (8.1)	.154
30‐day mortality	12 (0.7)	56 (0.7)	1.000

*Note*: Numbers in brackets represent percentage.

The bold numbers are the ones the are satistically significant, i.e. p‐value smaller than 0.05.

One‐year mortality rate was also not different between groups (Figure 1B, *p* = .36).

We analyzed clinical outcomes by dividing the study population into tertiles according to their GRACE risk score (see Supplementary Table 3). As expected, the rate of adverse events and mortality increased with increasing GRACE score. However, no statistically‐significant difference in outcomes was found between the short‐LOS and intermediate‐LOS groups except for the rate of re‐hospitalization which was higher in the highest GRACE score (>115) group (*p* = .03).

**FIGURE 2:**
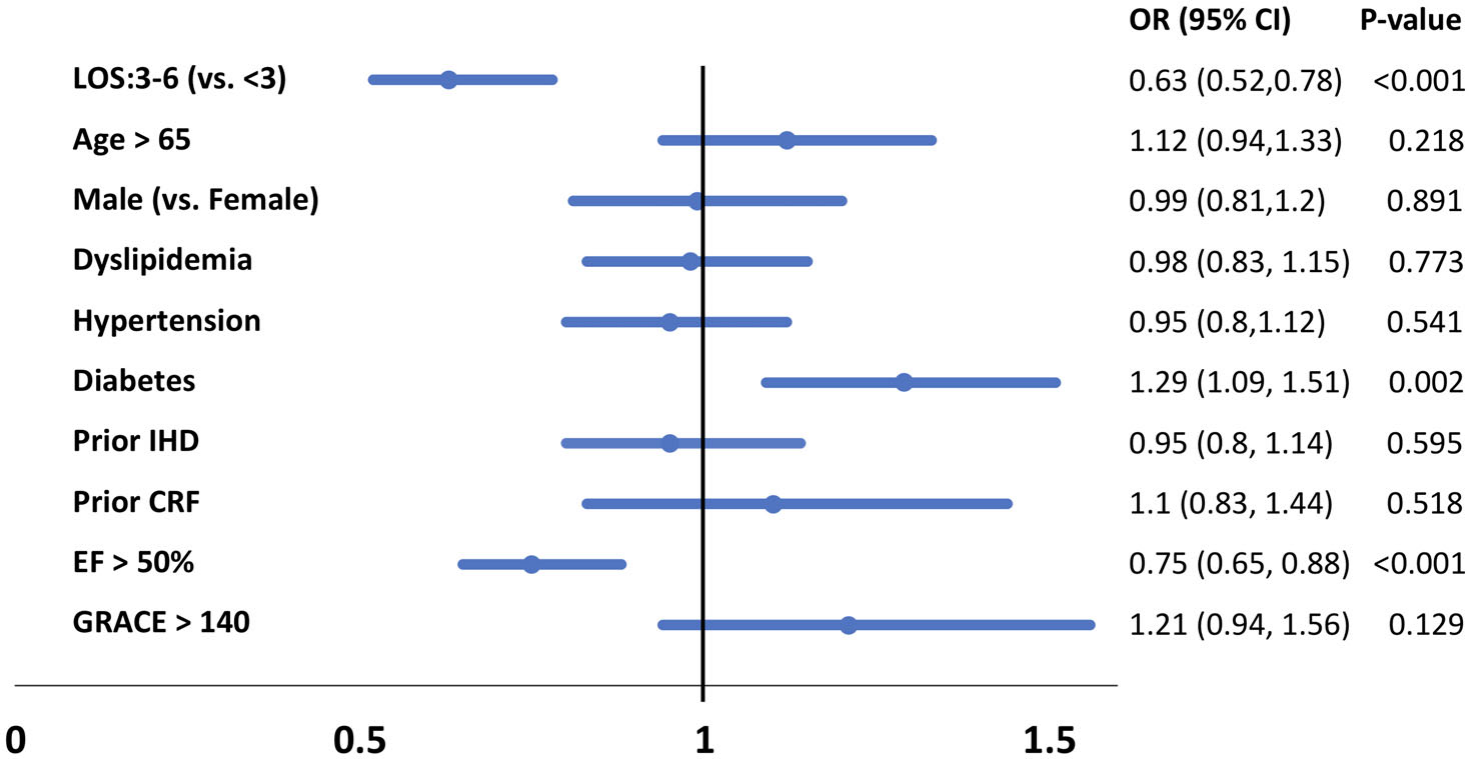
Multivariate analysis of predictors for re‐hospitalization in 30 days

We performed a multivariate analysis to identify significant predictors for re‐hospitalization within 30 days. Short length of stay, diabetes mellitus and a reduced ejection fraction were independently significant predictors for re‐hospitalization (Figure 2).

Logistic regression for the 30‐day MACE was performed using selected co‐variables. The 30 days MACE was assessed both in all patients and among STEMI patients separately (Table 3).

**TABLE 3:** Logistic regression for MACE at 30 days

Variable:	All patients HR (CI)	STEMI only HR (CI)
Hosp. duration: 3–6 (vs. <3)	1.10 (0.79,1.56) *p* = .59	0.75 (0.46,1.28) p = .27
Age (per year)	**1.03 (1.01,1.04) *p* < .0001**	**1.03 (1.01,1.05) p = 00005**
Male	1.02 (0.77,1.37) *p* = .89	1.03 (0.67,1.62) *p* = .89
Dyslipidemia	0.80 (0.63,1.03) *p* = .08	0.78 (0.55,1.10) *p* = .16
Hypertension	0.93 (0.72, 1.2) *p* = .58	0.78 (0.55, 1.14) *p* = .21
Diabetes	1.12 (0.88,1.43) *p* = .35	1.22 (0.84,1.75) *p* = .29
Prior IHD	1.23 (0.95,1.59) *p* = .11	1.55 (1.06,2.26) *p* = .03
Prior CRF	1.15 (0.77,1.67) *p* = .49	0.78 (0.33,1.61) *p* = .54
Normal EF (>50%)	**0.75 (0.60,0.94) *p* = .02**	**0.58 (0.40,0.83) *p* = .004**
GRACE >140	0.81 (0.56,1.16) *p* = .26	0.71 (0.33,1.42) *p* = .35
Observations	4930/9593	2500/4212

Abbreviations: CI, confidence interval; GRACE, Global Registry of Acute Coronary Events Risk; HR, hazard ratio.

Length of stay (short compared to intermediate length) was not associated with MACE at 30 days, both in all MI patients and in STEMI patients alone. The strongest predictors for MACE at 30 days were age and LV systolic function. Logistic regression analysis for 30 days mortality (data not shown) showed higher mortality for patients with higher GRACE score (HR 3.01, CI 1.09–8.61) and low EF (HR for normal EF: 0.23, CI 0.08–0.55) and a trend for older age (HR 1.04, CI 1–1.08). No other variables including LOS were significantly associated with mortality.

## 4 DISCUSSION

In the present study, we examined trends of hospital discharge of patients with MI since 2000 and assessed whether differences in LOS were associated with outcomes.

Consistent with general global trends,^6^ the LOS after acute MI gradually decreased throughout the years in our cohort. We examined whether very early discharge strategy, 3 days or less from admission, is safe for low and intermediate‐risk patients. Patients with long LOS (more than 6 days) were excluded as they were clearly older with more co‐morbidities (Table 1), had a more complicated course and therefore were not eligible for early discharge based on clinical judgment. Hence, we focused on the comparison between the short‐LOS (≤3 days) and intermediate‐LOS (4–6 days) patient groups as done previously in similar studies.^21^

The vast majority of these patients had an uncomplicated clinical course and in many cases stayed in hospital for observation as part of conservative and traditional policies. Indeed, we found that both groups had comparable baseline characteristics (Table 1). Surprisingly in the short‐LOS group there were more patients with risk factors and known ischemic heart disease. This may be due to a smaller need for evaluation time and medical adjustments for already medically treated patients or because patients with recurrent events are more familiar with the procedures and treatments and consequently do not require long hospital stay.

The short‐LOS group consisted of patients who were in slightly better clinical condition upon admission with fewer in‐hospital complications (which were low in both groups). More importantly, the clinical outcomes at 30 days as well as the 1‐year mortality were not different between the groups. Nevertheless, the rate of re‐hospitalizations was higher in the short‐LOS group but without evidence of cardiovascular events. Same results of higher rate of re‐hospitalizations after short LOS were noted in previous studies.^22^ The risk for re‐hospitalization correlated with the GRACE risk score and was very low for patients with GRACE score under 115. The rate of re‐hospitalization decreased throughout the years of the study, and correlation with short‐LOS became non‐significant in the later years (2010–2016). Re‐hospitalization was more common in older patients and in patients with diabetes mellitus and a reduced ejection fraction. This may indicate that a more conservative approach for these sub‐populations is warranted.

Our study supports current guidelines advocating discharge 48–72 h after admission for an uncomplicated STEMI.^8^ However, there is inconsistency in the literature about the optimal score for assessing the risk after MI in order to determine the desired length of stay. Most studies defined the threshold for early discharge as an extremely low risk in STEMI patients.^9^, ^11^, ^12^, ^15^, ^16^, ^23^ Our study incorporated a much wider spectrum of patients by the GRACE risk score, and demonstrated the same outcomes for both STEMI and NSTEMI patients at low to intermediate risk. When using a multivariate analysis, the LOS did not predict neither mortality nor MACE at 30 days (Table 3). Furthermore, there are no guideline recommendations for optimal LOS following NSTEMI^17^, ^18^, ^19^ and our study supports an approach of early discharge after an uncomplicated course.

### 4.1 Limitations

The major limitation of our observational study is that we cannot demonstrate causality but only an association between variables and outcome, along with the risk of overlooked confounders. In addition, the data was collected over a 16‐year period during in which various changes in therapies and intervention policies occurred that may have influenced the changes in LOS and prognosis.

The data were collected by many investigators, which may lead to different interpretations of the study questionnaire. In addition, re‐admissions were not divided into emergent and elective, and therefore may include some elective admissions such as planned PCI to a non‐culprit vessel. Finally, we could not account for patients admitted for very short time because of leaving against medical advice, a factor that may also portend worse outcomes secondary to noncompliance.

## 5 CONCLUSIONS

Our study suggests that an early discharge strategy of up to 3 days from admission is safe for low and intermediate‐risk STEMI and NSTEMI patients (GRACE score <115). The cause of increased re‐hospitalization in some populations remains unclear and might be related to non‐medical conditions. Potentially, more patients may benefit from an early discharge policy and shorter hospital stay, a policy that may be particularly relevant during the COVID‐19 pandemic.

## Supporting information


**Figure 1 Supplementary:** Percentage of patients in each group of length of hospital stay (LOS) throughout the years.

**Appendix S1.** Tables.

## Data Availability

Data available in article supplementary material.
