# Supplementary figures and images for: The impact of short hospital stay on prognosis after acute myocardial infarction: An analysis from the ACSIS database
Source: Clin Cardiol. 2021 May 26;44(6):748–53. doi: 10.1002/clc.23652 (PMC8207980; doi:10.1002/clc.23652)

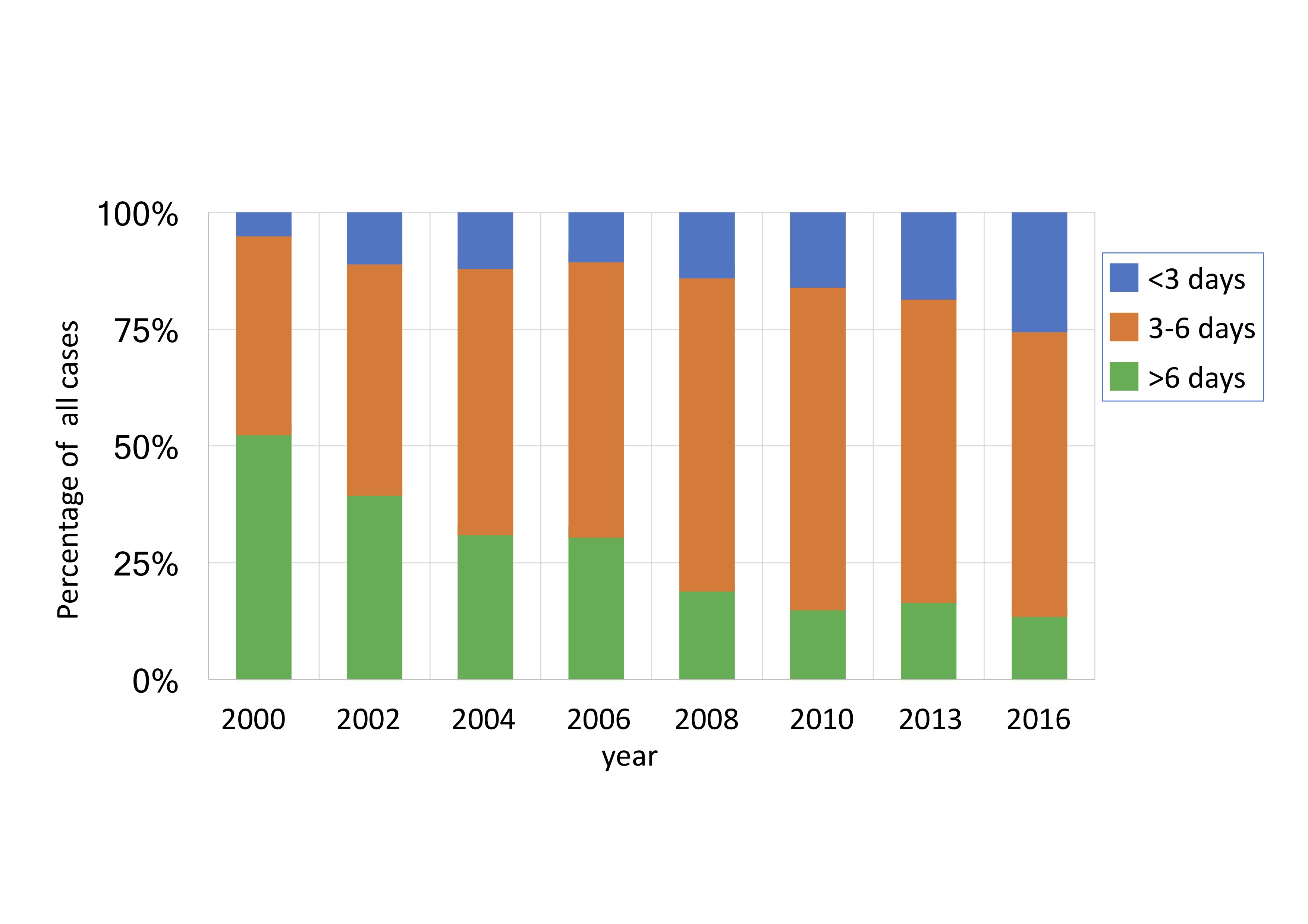

Supplement: Supplementary file 1 — Figure 1 Supplementary: Percentage of patients in each group of length of hospital stay (LOS) throughout the years. [file CLC-44-748-s001.jpg]
