# Supplementary material for: The impact of short hospital stay on prognosis after acute myocardial infarction: An analysis from the ACSIS database
Source: Clin Cardiol. 2021 May 26;44(6):748–53. doi: 10.1002/clc.23652 (PMC8207980; doi:10.1002/clc.23652)
Supplement: Supplementary file 2 — Appendix S1. Tables. [file CLC-44-748-s002.docx]

**Supplementary Material**

| Supplementary Table 1 **-** Clinical characteristics and hospital course stratified by hospitalization duration | | | |
| --- | --- | --- | --- |
| \|  \| **Hospitalization duration (days)** \| \|  \| \| --- \| --- \| --- \| --- \| \|  \| **<3** \| **3-6** \| **P value** \| \| N \| 1703 \| 7890 \|  \| \| **Clinical characteristics** \| \| \| \| | | | |
| Heart rate (bpm) (mean ± SD) | 78.31 ± 17.34 | 78.99 ± 18.88 | 0.174 |
| Systolic Blood Pressure (mmHg) (mean ± SD) | 143.48 ± 26.22 | 142.90 ± 27.76 | 0.433 |
| Diastolic Blood Pressure (mmHg) (mean ± SD) | 81.76 ± 14.76 | 82.05 ± 16.06 | 0.492 |
| AF/SVT | 48 (3.3) | 248 (4.0) | 0.207 |
| Normal ejection fraction (>50%) | 679 (62.1) | 3018 (49.0) | <0.001 |
| **Angiographic characteristics** | | | |
| Radial Vascular access (in PCI) | 123 (72.4) | 632 (56.1) | <0.001 |
| Infarct related artery (primary) |  |  | 0.635 |
| LAD | 101 (45.9) | 926 (46.9) |  |
| LCX | 32 (14.5) | 299 (15.1) |  |
| RCA | 82 (37.3) | 717 (36.3) |  |
| Number of diseased vessels (according to any angiography) |  |  | <0.001 |
| Single | 422 (33.8) | 2100 (37.6) |  |
| Multi | 727 (58.3) | 3221 (57.6) |  |
| None | 98 (7.9) | 265 (4.7) |  |
| **In-hospital complications** | | | |
| Pulmonary Congestion (Killip>1) | 90 (5.3) | 677 (8.5) | <0.001 |
| Post MI angina/re-ischemia | 31 (1.8) | 266 (3.4) | 0.001 |
| Primary VF | 10 (0.6) | 110 (1.4) | 0.009 |
| New A. Fib. | 23 (1.4) | 263 (3.3) | <0.001 |
| Acute renal failure | 27 (1.6) | 194 (2.5) | 0.036 |

Numbers in brackets represent percentage

| Supplementary Table 2 **-** Clinical outcomes at 30 days divided into early and late periods | | | | | | | |
| --- | --- | --- | --- | --- | --- | --- | --- |
|  | **Early Period (2000-2008)** | | | | **Late Period (2010-2016)** | | |
| **Hosp. duration (days)** | **<3** | **3-6** | **P value** | **<3** | | **3-6** | **P value** |
| N | 806 | 4776 |  | 897 | | 3114 |  |
| Re-hospitalization | 181 (22.8) | 882 (18.7) | **0.01** | 149 (18.9) | | 448 (16.2) | 0.08 |
| RE-MI | 11 (1.5) | 61 (1.5) | 1.00 | 8 (0.9) | | 34 (1.1) | 0.74 |
| Angina | 17 (5.0) | 95 (4.6) | 0.88 | 25 (3.3) | | 96 (3.5) | 0.81 |
| 30-day MACE | 80 (9.9) | 463 (9.7) | 0.89 | 40 (4.5) | | 177 (5.7) | 0.18 |
| 30-day mortality | 10 (1.2) | 38 (0.8) | 0.29 | 2 (0.2) | | 18 (0.6) | 0.29 |

*Numbers in brackets represent percentage

MACE - Major Adverse Cardiovascular Event, MI - Myocardial Infarction

| Supplementary Table 3 **-** Clinical outcomes at 30 days according to GRACE risk score tertiles | | | | | | | | |  |
| --- | --- | --- | --- | --- | --- | --- | --- | --- | --- |
| **GRACE score** | **Tertile-1 (<89)** | | | **Tertile-2 (89-115)** | | | **Tertile-3 (>115)** | |  |
| **Hosp. duration (days)** | **<3** | **3-6** | **P val** | **<3** | **3-6** | **P val** | **<3** | **3-6** | **P val** |
| N | 283 | 1856 |  | 319 | 1628 |  | 323 | 1751 |  |
| Re-hospitalization | 46(16.9) | 253(14.4) | 0.31 | 67(22.9) | 275(17.9) | 0.06 | 77 (25.3) | 327 (19.8) | **0.03** |
| RE-MI | 2 (0.7) | 16 (0.9) | 1.00 | 5 (1.6) | 27 (1.7) | 1.00 | 6 (1.9) | 34 (1.9) | 1.00 |
| Angina | 10 (4.6) | 54 (4.1) | 0.85 | 11 (4.3) | 40 (3.5) | 0.62 | 8 (3.5) | 37 (3.9) | 0.95 |
| 30-day MACE | 10 (3.5) | 95 (5.1) | 0.32 | 21 (6.6) | 120 (7.4) | 0.71 | 25 (7.7) | 166 (9.5) | 0.37 |
| 30-day mortality | 0 (0.0) | 1 (0.1) | 1.00 | 0 (0.0) | 9 (0.6) | 0.38 | 6 (1.9) | 25 (1.4) | 0.74 |

*Numbers in brackets represent percentage

MACE - Major Adverse Cardiovascular Event, MI - Myocardial Infarction
